# Patient perceptions of students in a longitudinal integrated clerkship in Taiwan: a qualitative study

**DOI:** 10.1186/s12909-021-02553-7

**Published:** 2021-03-10

**Authors:** Yaw-Wen Chang, David A. Hirsh, Wen-Hui Fang, Honghe Li, Wen-Chii Tzeng, Senyeong Kao

**Affiliations:** 1 Department of Family and Community Medicine, Tri-Service General Hospital, National Defense Medical Center, No.325, Sec. 2, Chenggong Rd., Neihu Dist., Taipei City, 11490 Taiwan, Republic of China; 2 Graduate Institute of Medical Science, National Defense Medical Center, No.161, Sec. 6, Minquan E. Rd., Neihu Dist.,, Taipei City, 11490 Taiwan, Republic of China; 3 Harvard Medical School Academy, Tosteson Medical Education Center, Room 384, 260 Longwood Ave, Boston, MA 02115 USA; 4 HMS-Cambridge Integrated Clerkship, Cambridge Health Alliance, 1493 Cambridge St, Cambridge, MA 02139 USA; 5 Institute of International Healthcare Professionals Education and Research, China Medical University, No.77, Puhe Rd., Shenyang North New Area, Shenyang, Liaoning P.R. China; 6 School of Nursing, National Defense Medical Center, No.161, Sec. 6, Minquan E. Rd., Neihu Dist., Taipei City, 11490 Taiwan, Republic of China; 7 Graduate Institute of Life Science, National Defense Medical Center, No.161, Sec. 6, Minquan E. Rd., Neihu Dist., Taipei City, 11490 Taiwan, Republic of China

**Keywords:** Undergraduate medical education, Medical student, Longitudinal integrated clerkship, Relationship, Continuity, Patient centered, Patient perception, Professionalism, Empathy, East Asia, Qualitative study

## Abstract

**Background:**

Longitudinal integrated clerkships (LICs) are a model of clinical education growing rapidly in Western contexts. LICs use educational continuity to benefits students’ clinical learning and professional identity formation. Patient-centered care is a core component of medical professionalism in the West. To support patient-centered care, education leaders in Taiwan restructured clinical education and implemented the first longitudinal integrated clerkship in East Asia. We aimed to investigate patients’ perceptions of longitudinal relationships with the LIC students within Taiwan’s Confucian cultural and social context.

**Methods:**

We invited patients or their family members who were cared for longitudinally by a LIC student to participate in the study. Participating patients or their family members undertook semi-structured interviews. We analyzed data qualitatively using a general inductive approach to identify themes in the patients’ descriptions of their experiences interacting with the LIC students.

**Results:**

Twenty-five patients and family members participated in interviews: 16 patients and 9 family members. Qualitative analysis of interview transcripts identified three themes from patients’ experience receiving care from their LIC students: care facilitation, companionship, and empathy. To provide care facilitation, LIC students served as a bridge between the physicians and patients. Students served patients by reminding, consulting, tracking disease progression, and researching solutions for problems. To provide companionship, students accompanied patients interpersonally like a friend or confidant who listens and provides a presence for patients. To provide empathy, patients reported that students showed sincere concern for patients’ experience, feelings, and mood.

**Conclusion:**

In our study, Taiwanese patients’ perspectives of LIC students suggested the value of care facilitation, companionship, and empathy. We discuss these themes within the context of Confucian culture and the Taiwanese context of care.

**Supplementary Information:**

The online version contains supplementary material available at 10.1186/s12909-021-02553-7.

## Background

Education leaders create longitudinal integrated clerkships (LICs) to advance students’ clinical and scientific learning and support their professional identity formation [1–5]. In contrast to traditional block rotations, LICs restructure clinical education to provide students’ educational continuity—continuity of care, curriculum, and supervision [5–8]. Through continuity, LICs reshape learning relationships and the learning environment [9, 10]. The literature on LICs reports educational benefits [4, 5, 11], and LICs support humanistic professional identity formation [12, 13], increase patient-centeredness [1, 3, 11, 14], and may improve communication skills [5, 15, 16]. Nonetheless, LIC research has been conducted primarily in the US, Canada, Europe, and Australia [17, 18].

The literature describes Western and East Asian conceptualizations of medical professionalism [19–22]. In these contexts, patients’ experience of care relates to patients’ self-concepts and patterns of self-other relationships, that are primarily culturally determined [23]. Authors describe differences between cultures even as commonalities exist across Western and East Asian contexts, heterogeneity exists within contexts, and both Western and East Asian conceptualizations are dynamic and changing [24]. In Taiwan and Mainland China, the medical professionalism framework has developed on the basis of Confucianism. This framework emphasizes personal integrity, benevolence, and collective social relations [19, 25–27]. The literature suggests that, consistent with Confucian tradition, East Asian patients expect the physician-patient relationship and the physician’s role to involve greater physician paternalism and authority [24]. Compared with patients in the West, East Asian patients may be less inclined to question or express concerns to their physician [24]. Western contexts appear to more strenuously evince the values of patient autonomy and physician-patient “shared-decision making” [21, 24]. In addition, in East Asian contexts influenced by Confucianism, patients--and physicians--also expect the close involvement of, even deference to, the patient’s family [21, 24]. In East Asia, cultural shifts and policies now promote patient-centeredness, although change has been slow [24]. In this Confucian culture context, it remains unclear the degree to which cultural or institutional influences support or impede these changes [24]. The literature also reports social contextual factors, including the health care system, government investment in healthcare, health insurance, and patient health literacy, impact patient experience of healthcare [28, 29].

To support patient-centered care, education leaders in Taipei, Taiwan restructured clinical education and implemented a LIC in a tertiary teaching hospital. The program intended that longitudinal relationships in LICs would support patient-centeredness. We developed our study’s conceptual framework by considering the principle of educational continuity and the longitudinal relationships that underpin the LIC structure [6, 30]. We theorize that just as students experience patient-centered beliefs arising from longitudinal relationships [31], patients are also affected by their longitudinal connection to students [32, 33]. Although the LIC literature does report patients’ positive reports of having LIC students in Western countries [32–34], we are aware of no studies in the Asian context of patients’ experiences of LICs. In this study, we investigated patients’ perceptions of longitudinal relationships with the LIC students in the Taiwan’s Confucian cultural and social context.

## Methods

### Introduction of the Tri-Service General Hospital longitudinal integrated clerkship

In Taiwan, medical students undergo 6-year medical programs beginning after high school. In this study, the LIC students came from the medical school of the National Defense Medical Center (NDMC) located in Taipei, a city of 2.7 million inhabitants [35] At NDMC, medical students enter clinical clerkships in their 5th year, followed by sub-internships in the 6th year. The core clerkships begin in October and end in May, and include internal medicine, surgery, gynecology/obstetrics, pediatrics, psychiatry, family medicine, diagnostic radiology, and clinical pathology. These rotations take place in the Tri-Service General Hospital (TSGH), a tertiary teaching hospital located in Taipei City. The Tri-Service General Hospital longitudinal integrated clerkship (TSGH LIC) provides two clerkship structures: a traditional block rotation and an LIC. The LIC takes place in the first 6 months of the core clerkship (5th) year. All clerkships at TSGH are hospital-based, because that is the principal model care delivery at NDMC. The LIC is a blended-type LIC [18] that occurs predominantly in TSGH’s tertiary inpatient setting. Medical students join this program voluntarily and are selected by lottery. Approximately 12 students enter this program each year (about 10% of the class).

The program pairs every LIC student with preceptors from each of the following disciplines: internal medicine, surgery, obstetrics/gynecology, pediatrics, psychiatry, and family medicine. The first 8 weeks of the LIC program are the inpatient immersion stage, during which the students are required to rotate on the wards of internal medicine, surgery, pediatrics, and gynecology/obstetric every 2 weeks. In the four months following the immersion, the students begin the integrated clinical learning and learning with their preceptors of six disciplines simultaneously. In this stage, the students primarily work with inpatient care teams and follow their patients into outpatient clinics. Preceptors assign longitudinal care patients to the LIC students based on the learning objectives and the patients’ conditions. The students will follow their patients by telephone or communication apps while patients are not hospitalized. They complete the follow-up through the course of patients’ treatment or until the conclusion of the LIC program.

### Study design and sample

We performed a qualitative study based on semi-structured interviews with patients or their family members who were being cared for and tracked longitudinally by LIC students in the academic year 2016–17. Interviews occurred between March to June 2017. We reviewed students’ patient logs, and invited all patients or their family members who fulfilled the following criteria to participate in this study: (1) the patient was cared for by a LIC student during the patient’s hospitalization; and (2) the patient had 3 or more contacts with a LIC student during an outpatient clinic, emergency visit, or rehabilitation after the hospitalization. If a patient was under 18 years old or had problems with verbal communication, consent was provided by the patient’s legal guardian, and this caregiver or parent was interviewed instead of, or along with, the patient.

The institutional review board of TSGH approved this study (TSGHIRB 2–105–05-050).

### Data collection

A trained research assistant, with no connection with the LIC program, conducted face-to-face interviews with LIC students’ patients or their family “guardian” using a predetermined semi-structured interview guide (Additional file 1). The research assistant asked the interviewees to describe how medical students were involved in the care process and how the patients felt about the involvement of the medical students. The research assistant transcribed the audio recordings verbatim and de-identified the transcripts. The primary investigators cross-checked the verbatim transcripts with the content of the original sound recordings to ensure the accuracy of the transcripts.

### Data analysis

We employed a general inductive approach to analyze the verbatim transcripts [36, 37]. We sought to identify themes that might emerge from patients’ experience of receiving care from their LIC students. Two primary investigators (YC, director of LIC program and WF, a preceptor the LIC program) first read 3 to 5 interview transcripts and separately performed the initial coding. Subsequently, they compared their work, discussed each other’s codes, and formed the initial codebook. In the next step, the two primary investigators and a research assistant coded another 3 to 5 interview transcripts according to these codes. Using the same process of deliberation and integration, these three researchers examined and discussed the codes, and refined the codebook. Hereafter, the primary investigators (YC, WF) and the research assistant coded the remaining interview transcripts using the coding instruction manual; the research assistant read and coded all the interview transcripts, whereas the two primary investigators each read and coded half of the interview transcripts. Thereby, each interview transcript was co-completed by the research assistant and one of the primary investigators. After completing the initial coding process, the two primary investigators (YC, WF) iteratively inspected and discussed all codes and categories, and generated the themes. In the final step, the primary investigators explained the themes and examples to all members of the research team (DAH, HL, WT, SK); the team then examined and discussed these results together to establish consensus.

## Results

The LIC students’ patient logs listed 57 longitudinal care patients who met the study’s two inclusion criteria. All patients were invited to be interviewed. The final study sample consisted of 20 patients. These patients comprised 25 interviewees: 16 patient interviewees and 9 family members (Table 1). Respondents under 18 years old were interviewed with or substituted by at least one parent or guardian. Patients in the final sample included recruits from all 12 students. All interviews were conducted face-to-face. The average length of interviews was 50 min (range 43 min to 68 min). Twenty-two patients turned down the interview invitation; 12 could not be reached by telephone, and 3 passed away. Of the 25 interviewees, 18 (72%) were female, and 7 (28%) were male. There was no significant difference in the proportion of females between participants (patients or their participating family members) and patients not interviewed (*P* > .05 for each comparison). The interviewees had an average age of 41.6 years; the youngest was 13 years old and the eldest 73 years old. There was no significant difference in the distribution of age between participants (patients or their participating family members) and patients not interviewed (*P* > .05 for each comparison). Other than sex and age, we did not collect information from patients who declined to participate.

**Table 1:** Identity and sex of the interviewees

	n(%)	Interviewee label
Interviewee identity		
Patient	16 (64%)	P002, P003, P004–1, P005, P006, P007, P008, P009–1, P011–1, P012–1, P013, P014, P015, P017, P018, P019
Family member	9 (36%)	P001–1, P001–2, P004–2, P009–2, P010, P011–2, P012–2, P016, P020
Sex		
Male	7 (28%)	
Female	18 (72%)	
Age	41.6 ± 15.7 years old	

**Fig. 1:**
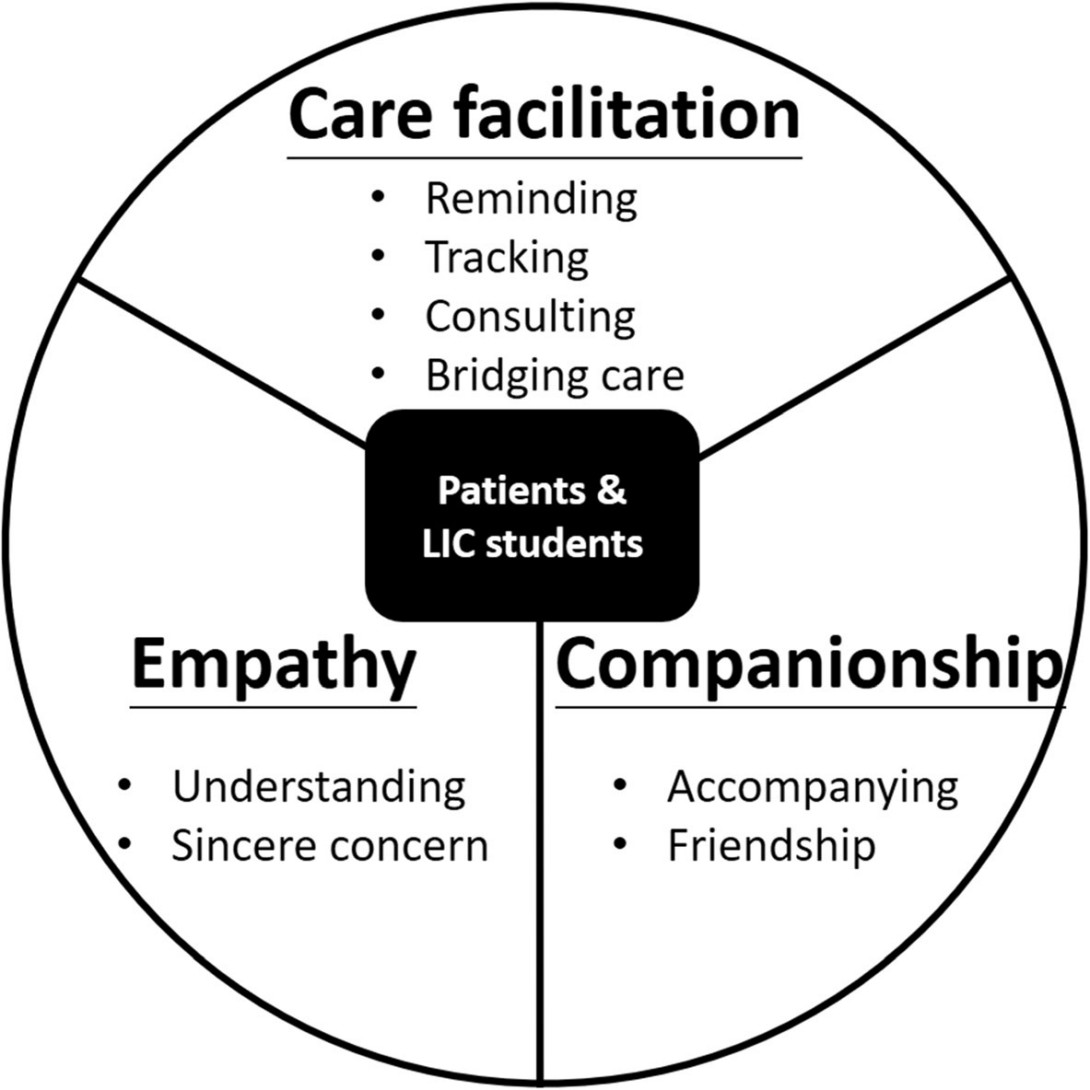
Major themes from patients’ descriptions of students’ participation in their longitudinal clinical care

We identified 3 themes from the patient interviews: care facilitation, companionship, and empathy (Fig. 1); we present themes and representative quotations for each theme. We summarize the findings below:

### Care facilitation

The interviewees recognize the student as a resource, connecting patients to their attending and improving patients’ access to the healthcare system. They mentioned their interactions with LIC students during hospitalization periods or at outpatient clinic sessions. The medical students kept in contact with the patients with telephone or instant messaging app. They reminded and confirmed the appointment date with patients, tracking their response to treatment, provided health education, and facilitated their care.

*“The medical student will LINE (a chat app) me and confirm the date of visit.” (P018)*

*“We chatted on the LINE. The students taught me how to control my diet in order to lower my blood sugar.” (P014)*

The interviewees described their student as a sort of consultant alongside the attending physician. The student not only provided consulting information but also offered them additional information about the hospital.

*“I will ask him (the medical student) which pediatrician is better at allergy or what. He is quite clear. He knows what the expertise of each doctor is.” (P002)*

*“In addition to the attending physician, you have the option of consulting other people, so you get the feeling of having a consultant team.” (P003).*

They expressed that LIC students strengthened the bridge between the attending physician and the patient. When patients would raise questions, the students provided answers directly or facilitated connection to the attending physician.

*“It is not easy to find my doctor. I can ask them [students] to help me ask. It may take some time, but I always get solutions to my problems.” (P005)*

*“They pass the message on to my attending physician. The physician then examines the situation and gives instructions.” (P001–1)*

### Companionship

The interviewees perceive the students’ presence. The students visited them daily while they stayed in the hospital, met them while they came back to the outpatient clinic, and kept in contact with them by telephone or communication apps when they were home. Numerous interviewees revealed that the LIC students were like a companion with professional knowledge and skills. They noted that students spent considerable time accompanying patients.

*“If I come to the hospital or clinic, he [the medical student] will come to see me, as long as I come to the hospital, he will look after me.” (P015)*

*“When we have a problem, we first think of him (medical student). Because we can only see the doctor at a certain time, but we can contact him (medical student) anytime.” (P011–1)*

Interviewees shared that the students cared about their wellbeing and shared relevant knowledge with them. With the students’ company and assistance, they would be less worried and feel at ease. Sometimes, the patients described students like friends they can talk to.

*“I feel like [the medical student] is a good friend, a very good friend. She is very caring and gave me a caring feeling.” (P001–2)*

*“Sometimes, there are things you don't want to talk to your family, [ … ] don't want to talk to the doctor. Medical students are more like friends, and sometimes you want to talk to friends about something.” (P012–1)*

Some interviewees expressed that they expected to see the students when they went back to outpatient visits, and like meeting a friend, the connection offered benefit.

*“It likes that when my son was going to see a doctor, he seemed going to have an appointment with his friends. He will feel a little happier because friends will come to see him in the waiting room.” (P009–2)*

### Empathy

The interviewees perceive the students’ attend to the patients’ feelings. Some interviewees explained that through longitudinal care, the LIC students could more deeply consider the patients’ experiences of being sick and patients’ experience of the treatment process. They expressed that they valued students’ displays of empathy.

*“They probably have a better understanding of the stress of having a chronic disease after they have gained experience of such contact with us [patients or family members]. They will be more empathetic.” (P011–2)*

*“They have a sense of empathy; they know the physical and emotional discomfort we are facing, so they comfort us in their own ways and help us snap out of our bad moods.” (P006)*

The interviewees also shared that students could better learn the families’ experiences about caring for these patients. They noted that medical students could understand their distress and support them in hard times.

*“She [the medical student] is not only focusing on my son [the patient] but also concerning my feelings. I feel being looking after.” (P020)*

*“In the process of communicating with medical students, you gradually know their professional abilities, and then they also understand your suffering and your blind spots.” (P010)*

The interviewees expressed that they received sincere concern from the students that were beyond the standard clinical diagnosis and treatment process.

*“They don't make me think that I am a patient. They can chat with me and make me feel like a friend. Because they may be about the same age, they can understand my thoughts well”. (P014)*

*“I was discharged from the hospital, but my baby has not been discharged yet. The medical student promised me to see my baby every day and report to me my baby's condition.” (P008)*

## Discussion

In this qualitative study, we used an inductive approach to analyze interviews of LIC students’ longitudinal patients or their family members. We extracted three themes: care facilitation, companionship, and empathy. To provide care facilitation, LIC students serve as a bridge between the physicians and patients. Students serve patients by reminding, consulting, tracking disease progression, and researching solutions for problems. When providing companionship, students accompany patients interpersonally like a friend or confidant who listens and provides a presence for patients. To provide empathy, patients reported that students showed sincere concern for patients’ experience, feelings, and mood. To our knowledge, this is the first study characterizing patients’ perspectives of their engagement with longitudinal students in the Asian context.

Our study connects to prior studies in the Western context that examined LIC students’ experiences with patients. In our context, the patients recognized their students as a resource to facilitate care. Patients described the benefits when students connected them to their attending physician and improved access to the healthcare system. In earlier qualitative studies, similar findings arose from *students’* perspectives, whereas our similar findings arose from patient perspectives. Earlier work in the US by Ogur and Hirsh described that students’ longitudinal care transformed the student role and improved patients’ experience of care [31]. Similarly, Hauer et al. reported that the LIC structure affords students opportunities to function in a doctor-like role and transmit and share information between the providers and patients [38].

The Western literature also reports *patients’* perspectives of having LIC students in their care. Flick et al. described the growth in student-patient relationships that develop over time and the symbiotic nature of the relationship; students’ learning benefits from the longitudinal relationship and the LIC students’ patients reported subjective improvement in health outcomes [33]. Poncelet et al. reported patients with more severity of illness described their longitudinal students as undertaking a physician-like role more than other patients who encountered students [32].

In the East Asian context of our study, Confucianism has profound influences on the patterns of government, society, education, and family. At the individual level, Confucian ethics is characterized by the promotion of virtue “De (德)” which consists of the five concepts: benevolence/humaneness “Ren (仁)”, righteousness “Yi (義)”, proper rite “Li (禮)”, knowledge “Zhi (智)”, and integrity “Xin (信)”. At a societal level, Confucianism emphasizes the importance of the family and social harmony, which is the practice of collectivism and regulated by loyalty “Zhong (忠)”, filial piety “Xiao (孝)”, contingency “Jie (節)”, and righteousness “Yi (義)”.

We consider our findings within our East Asian cultural context. Among the three themes we extracted from our patients’ interviews, two themes connect closely to medical professionalism in Taiwan: companionship and empathy. We found that our patients valued students’ considerable time keeping their company and understanding their feelings and emotions. This finding aligns with the study in the US, demonstrating that patients deeply valued the therapeutic alliances built with LIC students through longitudinal relationships [33]. Flick et al. describe that continuous and patient-centered care is the foundation of this alliance [33]. Does the “therapeutic alliance” in the Western context connect to our findings of “companionship and empathy”? We consider possible cultural underpinnings of the two latter themes derived inductively from our analysis. Following research from Ho et al. [19, 39] who describe the cultural influences on professionalism in Taiwan, we suggest that our Taiwanese patients’ responses may be influenced by two sources: Confucian culture and perspectives on local health systems.

Confucianism underpins Taiwanese culture, the context where the interviewed patients received care [40, 41]. The theme of companionship may relate to a Confucian focus that emphasizes the relationship between individuals and their society. In Taiwan, consistent with Confucian tradition, people recognize, expect, and seek individuals and their collective group to rely on each other mutually [42]. This “collectivism” contrasts with elements of Western individualism—particularly in the US where the earlier studies arose [23]. This difference may inform the meaning of our interviewee’s responses. In an Asian context, the essence of the companionship expressed by the patients may relate to their appreciation when students “show deep presence” and connectivity, Confucian values that may be currently rare in the fast pace of Taiwanese medical practice. The interviewed patients from this cultural context described that the LIC student contributes to other forms of caring beyond the typical medical approach. We wonder if patients may sense that students fill—even restore—a societal role through their longitudinal relationships.

The theme of empathy may relate to the Confucian notion of “Ren (仁)”—the central ethical principle of Confucian culture, “equivalent to the concepts of love, mercy, and humanity [43].” Ren is classically defined by a framing familiar to Western traditions: “Do not do to others what you do not want done to yourself” (己所不欲 勿施于人) [44]. In the Asian tradition, Ren relates directly to the concept of empathy as one of its core elements. In our study, patients specifically expressed valuing the Ren shown by the LIC students.

These patients’ perceptions and expectations of the Taiwanese health system may also inform their responses. Currently, Taiwan is experiencing a severe physician shortage [45]; consequently, large medical institutions run at, and beyond, full capacity [46]. The care delivery system in Taiwan limits opportunities for longitudinal interpersonal relationships due to the pace of care. Outpatient clinics in tertiary hospitals are usually busy and crowded, and doctors may need to see 50 or more patients in a morning [47] (some specialists may see 30 patients in an hour [48]). Patients typically have very short visits with the doctor [48]. The patients’ short contact time with physicians undermines patient-physician rapport; doctors have less time to act as companions or show empathy. Patients and doctors in Taiwan seek longer visits and long-term relationships, a circumstance not unlike the West. However, the *meaning* patients ascribe to deficits in physician companionship and empathy may be different in the Taiwanese context where the relationship is influenced by a Confucian sense of duty rather than a transaction.

### Limitations

This study has limitations. Initially, students listed 57 longitudinally-tracked patients as eligible for this study, but only 20 patients (with their families, comprising 25 interviewees) agreed to be interviewed. We do not know the opinions of the patients who were not interviewed. It is possible that patients who did not agree to be interviewed may have different, even negative views, of longitudinal relationships with students. Our attending physicians determine which patients the LIC students follow longitudinally on the basis of the patients’ suitability for interaction with medical students; this pool of patients may not represent the views of a general patient population in this hospital or elsewhere. Among those sampled, 72% were women, and we do not know the influence of sex on the results. We also do not know if the age distribution of our sample or other unmeasured demographic features represent the general patient population. We recognize that in 5 cases, patients and their family members were interviewed together, and in 4 cases, only family members were interviewed; we do not know the influence or effect of family opinions on the results. Considering reflexivity, our two qualitative researchers who performed the inductive analysis have connection to the LIC program. Although we used standard approach to analyze transcripts independently, we cannot exclude the possibility that our perceptions and consensus process contain biases. We note that this LIC progresses for 6 months, and LICs of greater length might have different impact on student-patient relationships. Finally, this study took place in one tertiary hospital in the largest city in Taiwan, and although it is the first such LIC patient study in Asia, we cannot assume the transferability of the results across other contexts.

## Conclusion

Our study of patients’ perspectives of LIC students suggests the value of care facilitation, companionship, and empathy in this Taiwanese care context. The LIC program creates a structure for medical students to establish longitudinal relationships with patients. It is our hope that longitudinal student–patient relationships extend the possibilities of the medical student role in Asia and beyond.

## Supplementary Information

**Additional file 1.** Interview guide.

## Data Availability

The datasets generated during and/or analysed during the current study available from the corresponding author on reasonable request.

## Abbreviations

LIC: Longitudinal integrated clerkship
TSGH: Tri-Service General Hospital
