# Supplementary material for: Patient perceptions of students in a longitudinal integrated clerkship in Taiwan: a qualitative study
Source: BMC Med Educ. 2021 Mar 10;21:153. doi: 10.1186/s12909-021-02553-7 (PMC7944602; doi:10.1186/s12909-021-02553-7)
Supplement: Supplementary file 1 — Additional file 1. Interview guide. [file 12909_2021_2553_MOESM1_ESM.docx]

**Additional file 1. Interview guide**

1. Please describe how the LIC students came to join or participate in your (or your family member’s) care process.
2. Please describe how the students contacted or interacted with you.
3. Please describe the role of the LIC students?
4. In your opinion, how would the longitudinal participation of medical students in the care process affect your (or your family member’s) medical intervention?
